# Home and Neighborhood Physical Activity Location Availability among African American Adolescent Girls Living in Low-Income, Urban Communities: Associations with Objectively Measured Physical Activity

**DOI:** 10.3390/ijerph18095003

**Published:** 2021-05-09

**Authors:** Ann Pulling Kuhn, Alexandra Cockerham, Nicole O’Reilly, Jacob Bustad, Victor Miranda, Tatiana V. Loboda, Maureen M. Black, Erin R. Hager

**Affiliations:** 1 Department of Pediatrics, School of Medicine, University of Maryland, Baltimore, MD 21201, USA; apullingkuhn@som.umaryland.edu (A.P.K.); mblack@som.umaryland.edu (M.M.B.); 2 Cartographic Products and Services Branch, U.S. Census Bureau, Suitland, MD 20746, USA; ammoulden@gmail.com; 3 School of Social Work, Boise State University, Boise, ID 83725, USA; nicoleoreilly@boisestate.edu; 4 Department of Kinesiology, Towson University, Towson, MD 21204, USA; jbustad@towson.edu; 5 Baltimore City Department of Recreation and Parks, Baltimore, MD 21217, USA; vmiranda@baltimorecity.gov; 6 Department of Geographical Sciences, University of Maryland, College Park, MD 21043, USA; loboda@umd.edu; 7 Department of Epidemiology and Public Health, School of Medicine, University of Maryland, Baltimore, MD 21201, USA; 8 RTI International, Research Triangle Park, Durham, NC 27709, USA

**Keywords:** geographic information systems (GISs), physical activity, accelerometer, African American, adolescents

## Abstract

Based on the ecological model of active living, the neighborhood environment may relate to individual physical activity (PA) behaviors. The purposes of this study were to (1) generate a replicable neighborhood-level physical activity location availability score (PALAS) from data variables associated with physical activity among adolescents and adults, and apply this score to Baltimore City, Maryland, and (2) determine if relationships exist between PA and PA location availability. Geographic information systems (GISs) were used to create the PALAS. Using linear regression models, we examined relations between objectively measured PA among low-income, urban, predominantly African American adolescent girls (*n* = 555, 2009–2012 data collection), and the PALAS rating of their neighborhood environment (neighborhood PALAS) and their home neighborhood area (PALAS variables/subcomponents within 0.25 miles of the home). A PALAS map of the study area was created, illustrating neighborhoods varying in availability and variety of PA locations. After adjusting for confounders, a higher neighborhood PALAS (β = 0.10, *p* = 0.041) and the presence of a recreation center in the home neighborhood area (β = 0.46, *p* = 0.011) were associated with more minutes per day spent in moderate to vigorous PA. Policy makers and stakeholders should consider increasing access to PA locations as a strategy to promote PA among adolescent girls.

## 1. Introduction

The U.S. Department of Health and Human Services Physical Activity (PA) Guidelines for Americans recommend that children and adolescents participate in at least 60 min of moderate to vigorous PA (MVPA) daily [1]. Time spent in MVPA is inversely associated with cardiometabolic risk [2], overweight and obesity [3], and depression [4]. U.S. children and adolescents are not meeting PA recommendations in general [5,6,7], with African American adolescent girls at the highest risk for inactivity [8].

The built and natural environment (all aspects of surroundings, man-made and natural) may influence risk for obesity by creating a setting that supports or hinders activity [9,10]. The association between specific environmental characteristics and physical activity in childhood has been extensively studied [11,12,13,14]. For example, an increased number of PA facilities has been shown to be associated with more time spent in MVPA [15]. Environments shown to support PA among children typically enable recreational activities, including parks, recreation fields, and green space (open land covered in grass or vegetation that could potentially be used for PA) [16,17,18,19]. The availability of green space, recreational space (e.g., recreational fields, basketball courts, etc., including those located on school grounds), parks, and facilities that support PA has been linked to increased PA [20,21,22,23,24] among children and adolescents. Examining a combination of physical activity promoting aspects of the built and natural environment together, in a single construct, could clarify how multiple environment factors relate to individual-level health behaviors.

Geographic information systems (GISs) data and methods have been used to map aspects of the built and natural environment and to explore the relation between environment and PA using objective spatial measures [22,25,26,27,28,29]. Locations used for PA have been mapped to identify disparities in spatial accessibility. The limited PA locations in low-income communities put adolescents who live in low-income communities at increased risk for inactivity [21,30,31]. The lack of safe spaces for outdoor PA, based on a physical environment inventory and caregiver and child perceptions further contribute to limited PA opportunities in low-income, urban neighborhoods [32,33]. GIS methods can be used to identify neighborhoods with characteristics that increase the risk for inactivity, enabling policy makers and stakeholders to identify areas to be targeted for additional PA locations.

The ecological model of active living conceptualizes how the neighborhood environment relates to individual behaviors, including participation in PA [34]. The model posits that PA is influenced through four hierarchical domains (1—policy environment, 2—behavior settings, 3—perceived environment, and 4—intrapersonal). The absence of PA promoting opportunities in the higher-ranking domains (1–3), limits individuals’ ability to pursue PA in the intrapersonal domain (4). This study focuses on the second ranking domain (behavior settings), which includes access to the built environment (e.g., neighborhood, recreation, and schools) and natural environment (e.g., vegetated open space, parks, and green space) in low-income, urban communities, assessed in relation to objectively measured PA in predominantly African American adolescent girls.

Based on the ecological model of active living [34], the purpose of this study is two-fold. Our first objective is methodological—we sought to generate a replicable neighborhood-level physical activity location availability score (PALAS) constructed from data variables associated with PA and apply this score to neighborhood statistical areas (NSAs, developed by the Baltimore City Planning Department based on 278 recognizable city neighborhoods) in Baltimore City [35]. Second, we examined the relation between objectively measured PA among predominantly African American adolescent girls and the PALAS rating of their neighborhood environment (neighborhood PALAS) and the home neighborhood area (HNA, PALAS variables/subcomponents within 0.25 miles of home). We hypothesized that adolescents living in high PALAS neighborhoods (indication of greater availability of PA locations) would engage in more minutes per day of MVPA compared to adolescents living in low PALAS neighborhoods. We also hypothesized that adolescents with PA locations in their HNA, including government-owned PA locations, schools, recreation centers, or parks, would engage in more minutes MVPA/day than adolescents without nearby PA locations.

## 2. Materials and Methods

### 2.1. Objective 1 (Methodological): To Generate a Replicable Neighborhood-Level PALAS and Apply This Score to Baltimore City Neighborhoods

#### 2.1.1. PALAS Development

Nine PALAS component variables were identified for inclusion in the PALAS based on a literature review and input by a team of experts (Table 1). All variables, except private PA locations (group variable 5), were obtained, compiled, and modified from existing digital spatial data layers, spanning 2010–2013 from the Capital Improvement office inventories at the Baltimore City Department of Recreation and Parks [36]. The most current maps are publicly available [36]. Private PA location variables and subcomponents (variables 5a–5c) were created in 2014 through a double-blind search procedure using preidentified search terms in Google Maps [37]. Following the established procedure, two independent coders identified 208 and 180 private PA sites, respectively, with 131 concordant locations. Coders cross-checked the discordant locations to confirm if the location was open, operating, and located within the boundaries of Baltimore City through follow-up web searches and telephone inquiries. The coders agreed on a final count of 218 locations. All the variables used in the PALAS calculations, except green space (variable 1), had a proximity buffer of 0.25 miles applied. A green space buffer was generated, however it was ubiquitously distributed across NSAs, resulting in near-complete saturation, therefore we chose not to include the green space buffer variable. If a proximity buffer of any individual variable intersected an NSA, the NSA was designated as having access to the PA location. A distance of 0.25 miles was chosen for the proximity buffer as this is the average distance people are willing to walk for services [38,39].

GIS mapping tools were used to combine variables by NSA. The base map of Baltimore City used in this study divides the city into 278 NSAs [36]. The variables were dichotomized (presence or absence) for each NSA. The NSA received a score of one for each variable present, regardless of the number present (i.e., an NSA with ≥1 recreation centers would receive a score of 1). A sum score was created for the variables, with a range of 0–9 (Table 1).

#### 2.1.2. PALAS Map

**Figure 1:**
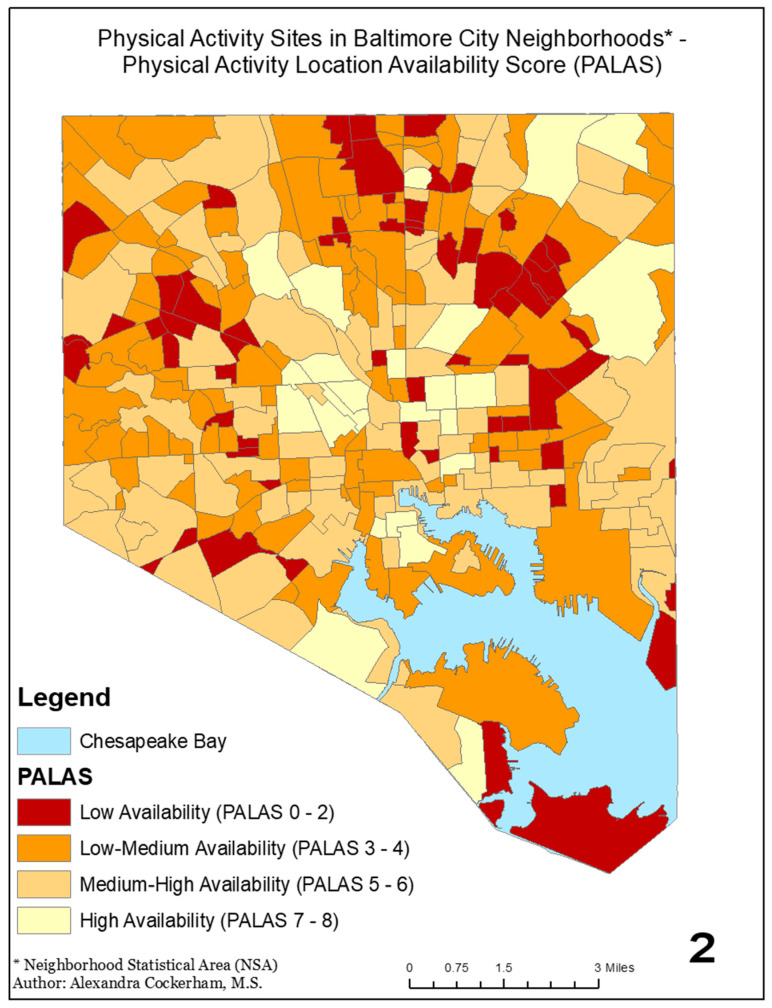
PALAS by neighborhood for Baltimore City (2014). PALAS: physical activity location availability score.

Figure 1 is a visualization of the PALAS for each NSA showing its distribution throughout Baltimore City. No NSA received a score of 9, making the highest score an 8. PALAS scores are ordinal, with a higher PALAS representing greater neighborhood access to PA locations.

### 2.2. Objective 2 (Applied): To Examine the Relationship between Objectively Measured PA among Predominantly African American Adolescent Girls and the PALAS Ratings of Both Their Neighborhood and the Area within 0.25 Miles of Their Homes

#### 2.2.1. Sample

Adolescent girls (*n* = 789) were recruited from 22 Baltimore City public schools serving predominantly low-income communities to participate in a health promotion study between 2009 and 2012. Eligibility criteria included female gender, enrollment in the 6th or 7th grade, ability to read and understand English, and successful completion of a brief health screen (able to participate in activities similar to those included in physical education class). Mean recruitment rate per school was 48% (range 22–85%). Caregivers and their adolescent daughters provided written informed consent and assent, respectively. The protocol was approved by the Institutional Review Boards at both the University of Maryland School of Medicine and the Baltimore City Public School System.

#### 2.2.2. Demographics

Adolescent girls self-reported birth date and race. Target population was predominantly African American or Black, therefore race was dichotomized as African American or Black, alone or in combination with another race versus not African American or Black. Transportation to/from school was also self-reported (dichotomized as does not walk to/from school versus walks to and/or from school).

#### 2.2.3. Anthropometrics

Height was measured to the nearest centimeter using a portable stadiometer (Shorr Productions, Olney, MD, USA) and weight was measured in kilograms using the TANITA TBF-300 body composition scale (TANITA Corp., Tokyo, Japan), both measured in duplicate to ensure reliable measurements. Gender-specific BMI-for-age percentiles (BMI z-score) were generated to describe the sample using CDC 2000 growth indices. A BMI z-score ≥ 85% was considered overweight/obese.

#### 2.2.4. Accelerometry

An Actical accelerometer was placed superior to the lateral malleolus of the non-dominant ankle using a hospital bracelet (once latched, the band cannot be removed unless cut off) and worn for at least 7 consecutive days without removal. Due to the waterproof nature of the Actical, the device can be worn while bathing or swimming without damage. Additionally, because of the small dimensions and light weight, the device can be worn while sleeping or playing sports (under a sock) without interference. Activity counts were collected in 1-min intervals and cleaned by removing the first and last days of partial data. We retained data for participants with at least one full, 24 h day and a maximum of 7 full, 24 h days of PA data. A validated threshold for MVPA when placing the Actical accelerometer on the ankle was applied to the data to determine time spent/day in MVPA [51]. Minutes per day in MVPA was not normally distributed (skewness = 2.25) and was normalized by taking the square root (skewness = 0.79).

#### 2.2.5. Neighborhood PALAS

Home addresses were geocoded using ArcGIS^®^ software by ESRI (Redlands, CA, USA). Geocoding is the process of determining the geographic coordinates of an address, and placing representative points on a map [52]. Each girl was assigned the neighborhood PALAS for the NSA of her residence. The sample resided in 130 of the 278 NSAs in Baltimore City (46.8%).

#### 2.2.6. PA Locations Near the Home

We compiled individual PA locations specified in the PALAS (see Table 1), located within each girl’s HNA (0.25 miles around the home). Green space within the HNA was not calculated due to its homogeneous distribution. The number of PA locations was dichotomized for each girl, with a score of 1 for the presence and score of 0 for absence of each category within the HNA.

#### 2.2.7. Data Analysis

Data were analyzed using SPSS 22.0 (IBM, Armonk, NY, USA). Significance was set at α = 0.05. Correlations and linear regression models were employed to examine relations between the PALAS and PA. *t*-tests and linear regression models were used to test for relations between PA locations in the HNA and PA. Age, BMI z-score, and walking to/from school were examined as covariates in linear regression models because: (1) PA decreases from childhood to adolescence [53,54,55,56,57,58], (2) PA differs by weight status among children and adolescents [59,60], and (3) walking to school encourages PA [61].

## 3. Results

### 3.1. Sample Description

Among the 789 girls recruited, 654 were randomized to receive an accelerometer during the evaluation and 560 had valid accelerometer data [51]. Reasons for missing accelerometer data include lost (*n* = 30), refused (*n* = 2), two or fewer days of data/no full 24 h period (*n* = 13), technical issues with set-up or download (*n* = 23), and no reason recorded (*n* = 24). Five addresses did not geocode (addresses reported were invalid), resulting in a final sample of 555 girls, representing 84.9% of the sample of 654. A sample size of 555 allows enough power (≥0.8) to detect an effect size of delta ≥ 0.12 to detect the slope difference from zero in the linear regression of PALAS in relation to MVPA. The delta is defined as the difference between the alternative and null values of the slope multiplied by the ratio of standard deviation of the covariate to that of the error term (STATA 16.1, Statacorp LLC, College Station, TX, USA). Given the nine PALAS possibilities, the power analyses suggest that a sample size of 555 will provide enough power to detect a small-medium effect between the nine PALAS and MVPA (effect size Cohen’s f ≥ 0.17) (GPower 3.0, Heinrich_Heine_Universitat Dusseldorf, Dusseldorf, Germany). The sample of girls included in the analysis did not differ from the sample excluded by age, race/ethnicity, or weight status. The sample is described in Table 2. The adolescent girls ranged in age from 10 to 14 years with a mean age of 11.67. The majority self-identified as African American or Black, alone or in combination with another race (89.9%), and half of the adolescent girls were overweight or obese (49.7%). Less than half (41.4%) of girls reported walking to/from school. The adolescent girls spent an average of 41.95 min in MVPA per day.

Table 3 describes the PALASs for the NSAs in which the girls in the analytic sample were living at the time of data collection. Among these participants, the average NSA PALAS was 5.10, (SD 1.67, range 0–8). About 6.8% of girls lived in neighborhoods with a low PALAS (i.e., 0–2), 31.2% lived in neighborhoods with a medium-low PALAS (i.e., 3–4), 37.2% lived in neighborhoods with a medium-high PALAS (i.e., 5–6), and 24.9% lived in neighborhoods with a high PALAS (i.e., 7–8).

Table 4 shows the percentage of girls with each PA location type present in their HNA. Government-owned PA locations were the most common; almost three-fourths of girls had at least one government-owned PA location present in their HNA (74.2%). The subcategories, school and other government PA locations, when evaluated separately, were both present in over half of the girls’ HNAs (52.3% and 59.8% respectively). Parks were also present in over half of the girls’ HNAs (58.0%). Recreation centers were present in about one-third of the HNAs (32.1%). Private facilities were the least prevalent type of PA location in the HNAs (13.7%).

### 3.2. Neighborhood PALAS and Objectively Measured PA

Significant bivariate relations were observed between MVPA and age (Spearman r = 0.113, *p* = 0.008), and MVPA and BMI z-score (Spearman r = −0.117, *p* = 0.006), but not between MVPA and walking to/from school (Spearman r = 0.044, *p* = 0.307). The significant relations of age and BMI z-score with MVPA support their inclusion as covariates in the linear regression models. A bivariate positive correlation was observed between the square root MVPA and the PALAS (Spearman r = 0.105, *p* = 0.013). This relation was retained in a linear regression model adjusting for age and BMI z-score (Table 5). A higher neighborhood PALAS was associated with more minutes per day spent in MVPA. When the raw minutes per day spent in MVPA are used, each higher PALAS increment equates to 5 min/day more in MVPA.

### 3.3. PA Locations near the Home and Objectively Measured PA

Table 6 shows the comparison of PA locations near the home and PA. *t*-tests suggested that the presence of recreation centers, all-government, and school locations were each associated with more minutes per day in MVPA (square root). Parks and other sites considered were not related with MVPA. In adjusted linear regression models, the presence of a recreation center in the HNAs was the only PA location type related to higher MVPA. The presence of a recreation center is associated with an increase of 6 min/day of MVPA.

## 4. Discussion

To investigate the second ranking domain of the ecological model of active living, this study successfully generated a PALAS to map PA location availability in Baltimore City, MD by NSA and illustrated that neighborhoods varied in availability and variety of PA locations. The PALAS was then used to analyze if a relation existed between the PALAS and PA among predominately African American adolescent girls living in low-income, urban communities. Using individual variables and subcomponents of the PALAS, PA location availability around each girl’s home (HNA) was also examined in relation to PA. As a result, this study found that a higher neighborhood PALAS and the presence of a recreation center in the home neighborhood area were associated with more minutes per day in MVPA.

This study found that adolescents living in high PALAS neighborhoods engage in more minutes of MVPA per day compared to adolescents living in low PALAS neighborhoods, supporting our first hypothesis. This result supports the ecological model of active living, showing that the neighborhood environment is associated with individual behaviors. This finding also adds to existing literature showing a link between greater variety and availability of PA locations and PA among children and adolescents, specifically among African American adolescents living in low-income communities, an understudied group at risk for inactivity and obesity [62]. Furthermore, this study’s focus on adolescent girls is important given the gender-based PA disparity that consistently shows girls as having lower activity levels and higher body fat percentages than boys [63]. Our examination of relations between PA location access throughout the entire neighborhood (not just surrounding the home) and objectively measured PA is novel and has potential policy implications. The methods used to generate the PALAS map could be used by other jurisdictions to identify areas with limited access to PA locations. Demonstrating a link between PA and the PALAS among a population at increased risk for inactivity and obesity adds support for this method as a tool for stakeholders and policy makers. The information can be used for investment decisions for new PA locations or zoning regulations to support PA locations in neighborhoods with limited access.

This study demonstrated that adolescent girls living in low-income, urban communities with recreation centers in their HNA (within 0.25 miles of the home) engaged in more minutes of MVPA per day than adolescents who did not have recreation centers in their HNA, supporting our second hypothesis, the ecological model of active living, and the current literature [64,65]. We also hypothesized that all government-owned locations within the HNA would be associated with more PA, which was found in the bivariate analysis; however, in adjusted models the relation was no longer present. Additionally, the subcategory of government-owned school PA locations was significant in the bivariate analysis, but not in the adjusted model. Contrary to our hypothesis, parks showed no relation with PA in either bivariate analysis or adjusted models.

There are several possible reasons why we did not find strong support for relations between adolescent girls’ MVPA and access to government-owned and school PA locations and parks in this sample, yet did find a relation with access to recreation centers. First, recreation centers are more likely to provide child-specific programming, which was not measured in this study. Additionally, information was not gathered on the presence of joint-use agreements supporting community access to school grounds, which may be a barrier to accessing PA opportunities on school locations. Third, data on park quality, safety and programming were not gathered, which may moderate relations between access and MVPA [66,67]. A final potential reason for the lack of support for a priori hypotheses could be the population studied. We based our hypotheses on existing literature, which has had limited inclusion of African American adolescent girls from low-income, urban communities [62,68,69]. Findings from other studies may not generalize to this population. For example, our group examined if positive perceptions of the neighborhood environment surrounding schools or the aesthetic features of these neighborhoods (assessed via a driving audit) were related to PA among predominantly African American adolescent girls in low-income, urban communities [70]. Contrary to what is typically observed in suburban neighborhoods, we found that having an overall lower positive perception of the neighborhood environment surrounding the school and a greater density of displeasing neighborhood aesthetics (graffiti, broken windows, and abandoned homes) were each related to higher levels of activity, compared to the inverse. Taken together, findings from this study support access to government-owned PA facilities (recreation centers, schools, and other government-owned PA locations). Recreation centers, in particular, should be developed, supported, and maintained to promote PA among adolescents. Investment in PA locations is critical to increase PA, which is needed to maintain a healthy weight [71]. Additionally, the dynamics between PA access and PA of populations living in low-income, urban neighborhoods should be explored further.

This study had several strengths and limitations. One strength of this study includes the use of objectively measured PA, which eliminates self-reporting errors and bias. Another strength is the attempt to include PA locations across multiple jurisdictions and categories and create a database of PA locations for analysis that represented a complete range of possible locations used for PA. Private and publicly available PA locations were included. Every attempt was made to include a location only if it was open and operational. A limitation of the study is the inclusion of only predominantly African American adolescent girls in low-income, urban communities, which limits generalizability. The age range of 10–14 years old may also be considered a limitation as children’s PA outdoors may depend on the availability of a caregiver to provide supervision. Future studies utilizing a PALAS should include older children with increased independence and autonomy to interact with their environment. Additionally, the cross-sectional design prohibits establishing a causal relationship and prohibits the examination of the selection bias (when PA spaces are established first and healthier people with more economic capital relocate nearby, known as selection bias, or if the PA spaces were developed after healthier people with more economic capital were living there, known as causation) [72,73]. Physical accessibility, programming (fitness classes, soccer leagues, etc.), quality, or capacity of the PA locations were not included in this analysis, representing a limitation. These factors may inhibit or facilitate local residents’ use of PA facilities and in turn their PA. A limitation to our mapping procedure could be missing private PA locations undetectable by internet search, although we tried to include all private PA locations. Finally, the PALAS and associated map were developed based on data from 2010 to 2014 to align with activity data collected in 2009–2012. If others wish to use the Baltimore City PALAS map and shapefiles from this study for a current analysis, it will need to be updated; however, all methods are described herein.

Generating a replicable method for mapping PA location availability could be useful for future researchers and policy makers. The PALAS includes many structures that may have been in place for decades (including schools and parks), however other components of the PALAS such as private sites are more mobile. The PALAS could be used in other cities as an indicator of PA location availability and used over time to distinguish changes in infrastructure for PA. PALAS data in addition to policy and planning documents could be used to illustrate connections between the built environment and policies pertaining to parks and recreation, changes in demographics, and urban development [74]. An analysis of the relation between the PALAS, PA locations in the HNA, and other populations (children and adults of all ages, genders, and incomes) should also be conducted. To evaluate the selection bias, how/when/where PA locations change over time (retrospective and prospective) should be analyzed in relation to PA. This could determine the casual impact of PA locations and health promotion among residents, or whether healthy people move into areas with greater PA location availability. Finally, future studies should consider incorporating programs and/or quality of PA locations, and the expansion of the definition and categorization of parks and green space to include only developed and accessible land. By mapping and analyzing the programming, quality, and/or accessibility at PA locations, stronger linkages with PA may be established.

## 5. Conclusions

In conclusion this study developed a PALAS map of Baltimore City, using methods that are replicable for other jurisdictions. Access to PA locations in a neighborhood and near the home (in particular, recreation centers) was associated with more time spent in MVPA for low-income, urban, predominantly African American adolescent girls, supporting the ecological model of active living. Policy makers and stakeholders should consider increasing, not decreasing, spatially distributed access to PA locations as a strategy to promote PA.

## Figures and Tables

**Table 1:** Physical activity (PA) location availability variables.

	Variable	Threshold Definition		Description	References: Support for Inclusion
		NSA	HNA		
1	Green Space	Y(1)/N(0)	Y(1)/N(0)	Green space that could possibly be used for PA (includes institutional land, open urban land, deciduous forest, evergreen forest, and mixed forest)	[40,41,42,43,44]
2	Parks	Y(1)/N(0)	Y(1)/N(0)	Parks at least one acre in area and that have at least one feature (ballfield, basketball court, playground, tennis court, multiuse field, volleyball court, fitness stations, multiuse trails, and paths)	[20,45,46,47]
3	Recreation Center	Y(1)/N(0)	Y(1)/N(0)	All city-owned recreation centers	[20,45,48]
4	Other Government/City-Owned PA Location	Y(1)/N(0)	Y(1)/N(0)	All city-owned (school and non-school) PA locations (ice rinks, soccer pavilions, tennis courts, pools, school playground, park playground, multiuse fields/courts, basketball courts, ballfields, golf courses, volleyball courts, bocce ball courts, horseshoe pits, outdoor fitness stations, skate parks, kayak/canoe launch points, dog parks, specialty boxing center, and specialty recreation facility) (see 4a–4b for more detailed lists)	[20,45,48]
	4a School-Owned PALocation	--	Y(1)/N(0)	All school-owned PA locations (playgrounds, ice rinks, multiuse fields, basketball court, etc.)	[20,45,48]
	4b Non-School Other Government/City-Owned PA Location	--	Y(1)/N(0)	All non-school-owned PA locations (ice rinks, soccer pavilions, etc.)	[20,45,48]
5	Private PA Location	Y(1)/N(0)	Y(1)/N(0)	All for-profit, non-profit, and residential PA locations (see 5a–5c for detailed lists)	[48,49,50]
	5a For-Profit PA Location	-	Y(1)/N(0)	All for-profit PA locations (gyms, health clubs, studios (gymnastics, yoga, pilates, boot camp, cross-fit, mixed martial arts, dance, karate, rock climbing, and roller blading), tennis courts, pools, fitness centers, ice rinks, golf courses [indoor and outdoor], skate parks, bowling alleys, and disc golf courses)	[20,49,50]
	5b Non-Profit PA Location	--	Y(1)/N(0)	All non-profit PA locations (Y of Central Maryland, community center)	[20,49,50]
	5c Residential PA Location	--	Y(1)/N(0)	All residential PA locations (gyms, fitness centers, health clubs, and pools)	[20,49,50]
6	Park Proximity Buffer *	Y(1)/N(0)	--	Parks at least one acre in area and that have at least one feature (ballfield, basketball court, playground, tennis court, multiuse field, volleyball court, fitness stations, multiuse trails, and paths)	[20,45,47]
7	Recreation Center Proximity Buffer *	Y(1)/N(0)	--	All city-owned recreation centers	[20,45,48]
8	Other Government/City-Owned PA Facility Proximity Buffer *	Y(1)/N(0)	--	All city-owned (school and non-school) PA locations (ice rinks, soccer pavilions, tennis courts, pools, school playground, park playground, multiuse fields, basketball courts, ballfields, golf courses, volleyball courts, bocce ball courts, horseshoe pits, outdoor fitness stations, skate parks, kayak/canoe launch points, dog parks, specialty boxing center, and specialty recreation facility)	[20,45,48]
9	Private PA Facility Proximity Buffer *	Y(1)/N(0)	--	All for-profit, non-profit, and residential PA locations	[49,50]

Abbreviations: PA = physical activity; NSA = neighborhood statistical area; HNA = home neighborhood area. * Buffer = 0.25 mile buffer intersects neighborhood.

**Table 2:** Sample description (*n* = 555).

	Mean (Range) or %
Age	
Years	11.67 (10–14)
Race/Ethnicity	
% African American	89.9%
Weight Status	
% overweight or obese	49.7%
BMI-for-age z-score	0.99 (−3.56–2.91)
Physical Activity	
Walks to and/or from school	41.1%
Minutes Per Day Spent in MVPA	41.95 (0–255.33)
Square Root (MVPA)	6.16 (0–15.01)

**Table 3:** Participant PALAS descriptives in Baltimore City.

	Percent of NSAs with (x = #) Value (%)	Cumulative Percent (%)
PALAS (x = 0)	0.7%	0.7%
PALAS (x = 1)	2.0%	2.7%
PALAS (x = 2)	4.1%	6.8%
PALAS (x = 3)	8.1%	15.0%
PALAS (x = 4)	23.1%	38.0%
PALAS (x = 5)	18.6%	56.6%
PALAS (x = 6)	18.6%	75.1%
PALAS (x = 7)	20.0%	95.1%
PALAS (x = 8)	4.9%	100.0%

**Table 4:** PA locations within 0.25 miles of girls’ homes (in HNA) (% present).

	Mean%
Private	13.7%
Residential (subset of private)	4.3%
Non-Profit (subset of private)	4.3%
For-Profit (subset of private)	7.9%
Parks	58.0%
Recreation Centers	32.1%
All government	74.2%
School government (subset of all government)	52.3%
Other government (not-school) (subset of all government)	59.8%

**Table 5:** PALAS and square root MVPA linear regression model.

	β (*p*)	95% CI
Intercept	1.921 (0.181)	−0.893, 4.736
PALAS	0.102 (0.041)	0.004, 0.200
Age	0.322 (0.006)	0.094, 0.549
BMI z-score	−0.191 (0.018)	−0.650, −0.033

**Table 6:** PA locations in the HNA *t*-test and linear regression model * Results with square root MVPA.

		Mean	t (*p*)	β (*p*)	95% CI
Private	Present	6.34	0.86 (0.39)	0.252 (0.305)	−0.229, 0.733
	Not Present	6.13			
Residential (subset of private)	Present	6.29	0.334 (0.74)	0.275 (0.509)	−0.543, 1.092
	Not Present	6.15			
Non-Profit (subset of private)	Present	6.87	1.788 (0.07)	0.663 (0.109)	−0.149, 1.476
	Not Present	6.13			
For-Profit (subset of private)	Present	6.23	0.243 (0.81)	1.199 (0.228)	−0.755, 3.153
	Not Present	6.15			
Parks	Present	6.22	0.85 (0.34)	0.412 (0.082)	−0.052, 0.875
	Not Present	6.07			
Recreation Centers	Present	6.51	2.846 (0.01) **	0.461(0.011) **	0.108, 0.815
	Not Present	5.99			
All government	Present	6.28	2.376 (0.02) **	0.438 (0.068)	−0.032, 0.908
	Not Present	5.82			
School location (subset of all government)	Present	6.34	2.27 (0.02) **	0.487 (0.140)	−0.161, 1.135
	Not Present	5.96			
Other government (not-school) (subset of all government)	Present	6.27	1.652 (0.10)	0.432 (0.062)	−0.022, 0.886
	Not Present	5.99			

* Adjusting for age and BMI z-score ** *p* > 0.05.

## Data Availability

The authors will post all shapefiles on the Baltimore Neighborhood Indicators Alliance (BNAI) website.
